# Modelling the impact of forest management and CO_2_-fertilisation on growth and demography in a Sitka spruce plantation

**DOI:** 10.1038/s41598-023-39810-2

**Published:** 2023-08-18

**Authors:** Arthur P. K. Argles, Eddy Robertson, Anna B. Harper, James I. L. Morison, Georgios Xenakis, Astley Hastings, Jon Mccalmont, Jon R. Moore, Ian J. Bateman, Kate Gannon, Richard A. Betts, Stephen Bathgate, Justin Thomas, Matthew Heard, Peter M. Cox

**Affiliations:** 1 Met Office Hadley Centre, FitzRoy Road, Exeter, EX1 3PB Devon UK; 2 Department of Mathematics and Statistics, Faculty of Environment, Science and Economy, University of Exeter, Exeter, EX4 4QE UK; 3 Forest Research, Alice Holt Lodge, Farnham, GU10 4LH Surrey UK; 4 Forest Research, NRS, Roslin, EH25 9SY Midlothian UK; 5 School of Biological Sciences, University of Aberdeen, King’s College, Aberdeen, AB24 3FX UK; 6 Department of Biosciences, Faculty of Health and Life Sciences, University of Exeter, Streatham Campus, Rennes Drive, Exeter, EX4 4RJ UK; 7 Land, Environment, Economics and Policy Institute (LEEP), Department of Economics, University of Exeter Business School, Exeter, UK; 8 University of Exeter Global Systems Institute, Exeter, EX4 4QE UK; 9 The National Trust, Heelis, Kemble Drive, Swindon, SN2 2NA UK

**Keywords:** Forest ecology, Climate and Earth system modelling

## Abstract

Afforestation and reforestation to meet ‘Net Zero’ emissions targets are considered a necessary policy by many countries. Their potential benefits are usually assessed through forest carbon and growth models. The implementation of vegetation demography gives scope to represent forest management and other size-dependent processes within land surface models (LSMs). In this paper, we evaluate the impact of including management within an LSM that represents demography, using both in-situ and reanalysis climate drivers at a mature, upland Sitka spruce plantation in Northumberland, UK. We compare historical simulations with fixed and variable CO_2_ concentrations, and with and without tree thinning implemented. Simulations are evaluated against the observed vegetation structure and carbon fluxes. Including thinning and the impact of increasing CO_2_ concentration (‘CO_2_ fertilisation’) gave more realistic estimates of stand-structure and physical characteristics. Historical CO_2_ fertilisation had a noticeable effect on the Gross Primary Productivity seasonal–diurnal cycle and contributed to approximately 7% higher stand biomass by 2018. The net effect of both processes resulted in a decrease of tree density and biomass, but an increase in tree height and leaf area index.

## Introduction

The least severe Shared Socio-economic Pathways (e.g. SSP126), used to project the climate into the twenty-first century, assume significant changes in land-use with agricultural land being replaced by forests across the globe^1^. In 2020 an estimated 42% of Nationally Determined Contributions (NDCs) for the mitigation of climate change reported to the United Nations under the Paris Agreement currently involve some form of afforestation and reforestation^2^. Most national estimates of the total carbon sequestered through forestry policies are produced using simple IPCC methodology^3,4^.

In many countries with intensely managed landscapes, most afforestation is likely to be in the form of planted and managed forests (i.e., not by natural regeneration) which have a well understood stand history compared with natural forests. For instance, conifer plantations established for timber production are commonly referenced within the context of afforestation potential in the UK^5^. These plantations are typically even aged stands, with standard initial tree densities, established thinning regimes^6^ and with well understood empirical relationships between total carbon and biomass against stand age^7^.

### Empirical modelling of afforestation within the United Kingdom

A report of the UK Climate Change Committee^8^ estimated that if the policy target afforestation rate of 30,000 ha^−1^ from 2025 was achieved, the net sequestration would rise by 12 MtCO_2_e yr^−1^ by 2050 (‘Headwinds’ minus ‘Business as Usual’ scenario). The models employed in such projections and in the national greenhouse gas inventory, such as C-FLOW^9^ and CARBINE^10^, use empirical species-specific forest stand growth rate curves^6,11^, information on stand ages and assumptions about thinning and harvesting regimes, and model soil carbon change. However, these models may not include the effect of changing stand growth rates caused by both changing climate and CO_2_ concentrations. Additionally, climate change is likely to cause more frequent disturbances such as drought, pests and disease, windthrow, and wildfire across the UK^12–16^. While there are newer empirical methods to account for increased risk to yields from disturbances^14^, it may be difficult to quantify overall associated mortality loss. Empirical models have been used to simulate diverse woodland^17^. However, these studies often assume fixed species composition as a stand matures, when there could be competitive exclusion towards more climate resilient species^18^. Finally, there are strong arguments for a more holistic approach to planning afforestation that attempts to capture both risks and benefits of changing land use for biodiversity, food production, health, and recreation^5^. Optimisation of afforestation only for carbon sequestration could overlook these key related dimensions for land use decision making.

### Afforestation within land surface models

In comparison to more empirical forestry models, Land Surface Models (LSMs) are arguably more comprehensive in their representation of the multiple dimensions that a researcher or policymaker may need to consider. For example, the Joint UK Land Environment Simulator (JULES) LSM simulates the surface water and energy balance^19^, along with the carbon cycle in the natural vegetation, crops^20^ and soil^21^. As JULES is used within the Met Office weather and climate models, it has been evaluated at multiple scales^22–24^. Recent developments have introduced forest demography into Dynamic Global Vegetation Models (DGVMs) for use in Earth System Models and LSMs allowing for greater realism at larger scales^25^. However, there are few evaluations of plant demography ^26,27^, even less for the implementation of managed forests in DGVMs^28^. The implementation of thinning and other forestry management practices may lead to very divergent responses compared to natural forest regrowth^29,30^.

Managed forests with fixed initial tree densities and well understood yield curves represent a suitable ‘control experiment’ for new demographic DGVMs to be evaluated against. For example, there is strong competition between individual trees for resources, which provides a useful constraint for demography models with varied implementation of canopy-competition dynamics^25^. At the same time, high-resolution re-analysis datasets of meteorological drivers offer new opportunities for comparisons between NDC inventories and DGVMs^31,32^. Forest plantations are particularly relevant to future policy for achieving Net Zero emissions targets and provide a useful situation to evaluate new demographic DGVMs within LSMs. Additionally, including forest management as a process in DGVMs could potentially help explain the discrepancy between NDCs and large biogeophysical modelling efforts such as the Global Carbon Budget^33–37^.

## Methods

In this study, we explore two potentially significant factors in afforestation: CO_2_ fertilisation and forestry management. CO_2_ fertilisation and forestry management typically lack representation in empirical forestry models and LSMs, respectively. To that end, we compare a demographic LSM against an empirical representation of stand-growth at an appropriate mature forest stand with historic management. This allows us to evaluate modelled processes against biomass growth, size-structure and carbon flux observations. The overall objective is to clearly demonstrate how different approaches to modelling forest dynamics, empirical models and biogeophysical LSMs, can benefit from each other.

### Site Selection

We utilise data from a well observed stand in Harwood Forest (55° 13′ 00.2″ N 2° 01′ 31.2″ W). This is a second rotation Sitka spruce (*Picea sitchensis* (Bong.) Carr.) plantation of 40 ha, established in 1973 with a yield class of 18 m^3^ ha^−1^ yr^−1^ (‘YC18’, representing the maximum average annual stem productivity) growing on peaty-gley soil at an elevation of 290 m with a 2° slope. An instrumented ‘flux tower’ was installed in 2013, and the impact of the UK 2018 summer drought on energy, carbon, water fluxes has previously been reported^38^. This study uses measurements of meteorological, energy and gas flux half-hourly observations during 2015–20. During this period the flux tower had a mean precipitation of 1352 mm yr^−1^ and a mean annual temperature of 7.8 °C. Half-hourly measurements were provided for net ecosystem exchange (NEE), gross primary production (GPP) and net ecosystem respiration. Measurements of soil respiration (soil CO_2_ emissions, including litter, roots, and soil heterotrophs) and leaf area index (LAI) were provided at intermittent times between 2015 and 2021. Importantly for this study, the size structure was recorded in 2018 by measuring tree diameter at breast height (dbh) greater than 7 cm, to the nearest cm, in ten 200 m^2^ plots.

We infer half-hourly observations of tree respiration and net primary productivity (NPP). Firstly, we interpolate soil respiration at a half-hourly timestep by using a Q10 temperature-respiration function, using the nearest observed soil respiration measurements and the mean temperature for the observed soil respiration interval. Secondly, for the contribution of the roots to total soil respiration, we assume a value of 42% with an uncertainty range of 30–50%. This covers the spread of values seen geographically globally^39,40^. Thirdly, by taking the difference between the total ecosystem respiration and the inferred non-root soil respiration we estimate the tree respiration. Therefore, taking the difference between the GPP and the inferred tree respiration provides an estimate for the total NPP of the stand.

For estimating the height ($h$), carbon mass ($m$) distributions and total carbon stock of the forest we rely on allometric relationships. For tree height, we adapt the uniform height curve for even-age stands from Arcangeli et al.^41^ as shown by Eq. (1):

1 $$\begin{array}{c}h=1.3+\mathrm{exp}\left\{a+\left(\frac{-7.55}{\mathrm{dbh}}\right)\right\}.\end{array}$$

we use $a=3.17$ to give the maximum observed height of 25 m. For estimating the tree carbon mass (*m*), we adapt the allometry suggested by Black et al.^7^ for estimating dry mass in Eq. (2):

2 $$\begin{array}{c}m= 0.5\times \left[0.286\times {\left(\mathrm{dbh}\times \mathrm{h}\right)}^{1.138}\right],\end{array}$$

where the factor of 0.5 represents the approximate ratio of carbon mass to total dry mass for a tree.

### Simulations setup

Six simulations were carried out: fitted demography was used with in-situ measured meteorological forcing data (2015–20) and using regional daily climatic data^31^ (2015–17), and four longer period ‘historical’ simulations (1973–2017), which modelled the time-course of stand development from an initial planting density of 2500 trees ha^−1^. The four historical simulations controlled for forest management and transient CO_2_; thinned and unthinned with historical transient CO_2_ concentrations, thinned and unthinned with fixed 1973 CO_2_ concentrations. The specifics of each simulation, including the initial demography, are described in Table 1. We use a demography representing LSM called JULES-RED (Robust Ecosystem Demography)^42^ that includes a simple implementation of forestry management and a new implementation of canopy-closure (See ‘Supplementary Information—JULES-RED Model Description’).

**Table 1:** JULES-RED simulations conducted in the study.

Simulation name	Initial demography	Forcing	Period	CO_2_	Thinning
Fit. Dem., F.T	Fitted	Flux Tower	2015–2020	Transient	Yes
Fit. Dem., C	Fitted	CHESS-met	2015–2017	Transient	Yes
Hist. Th.	2500 trees ha^−1^	CHESS-met	1973–2017	Transient	Yes
Hist. Th., F. CO_2_	2500 trees ha^−1^	CHESS-met	1973–2017	1973	Yes
Hist. Uth.	2500 trees ha^−1^	CHESS-met	1973–2017	Transient	No
Hist. Uth. F. CO_2_	2500 trees ha^−1^	CHESS-met	1973–2017	1973	No

First column gives the simulation name/code source; Fitted Demography at Flux Tower (Fit. Dem., F.T.) and with CHESS-met (Fit. Dem., C.), Historical (Hist.) simulations: Thinned (Th.) and Unthinned (Uth.) with or without Fixed 1973 CO_2_ (F. CO_2_). Initial Demography refers to the initial state of the number density across JULES-RED mass classes; ‘Fitted’ uses the 2018 observations of number density fitted onto mass classes, while ‘2,500 tree ha^−1^’ refers to an initial planting in the lowest mass classes in 1973. Period indicates the model run time. The simulated CO_2_ forcing is described either as ‘Transient’ being historical CO_2_ and ‘1973’ is a fixed CO_2_ simulation with 1973 concentrations. If a simulation has thinning, a third of trees are removed after 25 years since simulation start time. As there is evidence of previous thinning at the Harwood site, fitted simulations are counted in this category.

For the fitted demography runs with the in-situ and CHESS-met climate data we use a Gaussian Kernel Density Estimator from the observed 2018 masses binned into the JULES-RED mass classes. For the historical CHESS-met simulations we initialised with 2500 trees ha^−1^ in the lowest JULES-RED mass class in 1973. This corresponds to a standard planting density for Sitka spruce for the UK^6,43^. To represent thinning, we track the stand age and after 25 years (i.e. in the year 1998 for the stand examined) we remove a third of trees uniformly across the size-structure. The thinned woody carbon being assumed to be used for non-decaying products, with the leaf and root carbon being added onto the local litter flux. This management regime is normal practice for first thinning of a second-rotation Sitka spruce plantation in the UK^44^. To represent Sitka spruce, we select the Needle-leaved Evergreen Tree (NET) Plant Functional Type (PFT) in JULES-RED as the closest approximation. The NET PFT was assumed to have a baseline mortality rate of 0.01 trees yr^−1^ and an assumed low rate of reproduction.

We compare historical simulations of JULES-RED against the UK Woodland Carbon Code (WCC) biomass lookup table^45^, which uses estimates of carbon sequestration from the CSORT empirical model in five-year periods of stand age for UK tree species^43^. The table output has been converted from units of tonnes of CO_2_ to tonnes of carbon (using a factor of 12/44). The WCC living biomass (hereafter ‘stand biomass’) and the total thinned carbon is derived from the cumulative sum of the stand carbon sequestration (WCC lookup table: ‘Carbon Standing’) and thinning rate (WCC lookup table: ‘Removed from Forest’) and the period duration. From the lookup table we selected both thinned and unthinned Sitka spruce YC18 planted at 2.0 m separation, which corresponds to an initial planting density of 2500 trees ha^−1^, as a comparison. The management employed in the WCC lookup table is more intensive than assumed in JULES-RED, with thinning occurring nearly every period beginning at the 15–20 year stand age interval.

### Simulation forcing and ancillaries datasets

For the historical simulations we used the Climate Hydrology and Ecology research Support System meteorology (CHESS-met) historical dataset (1961–2017) at 1 km resolution for the UK^31^. CHESS-met contains the necessary driving variables, radiative and meteorological, at daily time-steps. For simulations at the Harwood site, we used the nearest CHESS-met grid-box (centred approximately 160 m from the flux-tower). The Harmonised World Soil Dataset (HWSD)^46^ was used to infer the van Genuchten soil properties in JULES-RED at the UK CHESS-met spatial resolution^47^. For the prescribed historical CO_2_ concentration, we used the NOAA ESRL Mauna Loa Annual mean CO_2_ concentration 1960–2021^48^. By 2018, when the Harwood size-structure observations were recorded, the difference between the transient and 1973 concentrations was 79 ppm.

In addition, we also used the in-situ radiative and meteorological forcings from the Harwood flux tower. However, there were a few modifications necessary before running the simulations. Air pressure and wind speed had missing data within the time-series, which we linearly gap filled between the last and next observed values. As the specific humidity was only measured between 2015 and 19, we used the August-Roche-Magnus formula for the saturation vapor pressure, coupled with the relative humidity to fill in the missing data. The measured downward longwave radiation also appeared to have systematic errors. To resolve this, we used interpolated CHESS-met data for downward longwave for 2015–17 and extrapolated the average seasonal cycle to the end of 2020. Finally, downward shortwave radiation was truncated to zero to eliminate occasional small negative values at night.

## Results

### Evaluation of demography

**Figure 1:**
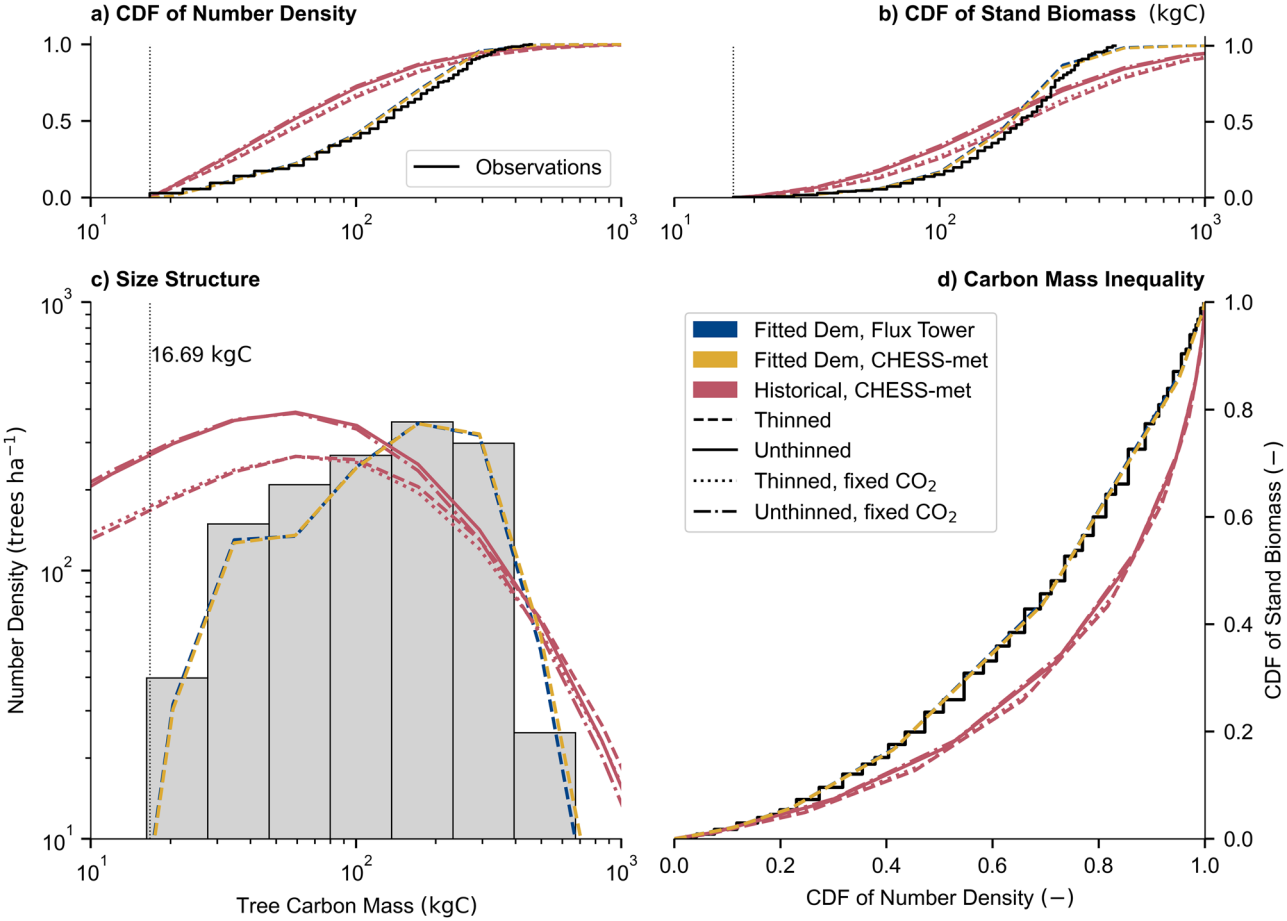
Observed and simulated 2018 demographic profile for the mature even aged spruce stand in Harwood Forest. Panels (**a**) and (**b**) respectively, show the Cumulative Density Function across tree mass from the truncation mass for the stand density and stand carbon stock. Panel (**c**) shows the distribution of trees across mass for JULES-RED and observations, where the observations have been binned into JULES-RED mass classes. Panel (**d**) shows the carbon distribution across the stand population or the ‘biomass inequality’ of the stand. The truncation mass (vertical dotted line) of 16.7 kgC is estimated by combining the minimum surveyed dbh of 7 cm with the allometric equations for estimating the tree carbon mass (see methods).

All historical simulations overestimated the number of measured small and large trees (Fig. 1c). The fitted observations of tree mass are indicative of the ‘best-case’ (lowest error) for the JULES-RED model. Implementing an assumed 33% evenly-applied thinning of trees after 25 years contributed to a reduction in the overall error between the observed mass distribution and JULES-RED (Table 2). The historical CO_2_ change resulted in a smaller reduction in the distribution error (difference in Chi-squared) compared with the observations. Implementing both thinning and CO_2_ fertilisation decreased the tree density by 24%, the biomass by 4.2%, and increased the height by 5.1% and LAI by 6.6%, compared to the unthinned and fixed CO_2_ simulation by 2018. However, these historical simulations all underestimated the mean tree height and LAI.

**Table 2:** The observed and simulated stand physical characteristics of Harwood in 2018.

Harwood 2018	N. Den	C. Den	Height	LAI	Gini	${\chi }^{2}$		KS test	
	ha^−1^	tC ha^−1^	m	m^2^ m^−2^		${\chi }_{\mathrm{dof}=6}^{2}$	$p$	$D$	$p$
Observations	1,348	208	17.6	5.56	0.36				
Fit. Dem., F.T	1,248	219	17.8	5.57	0.35	13	0.03	0.06	0.28
Fit. Dem., C	1,254	224	17.9	5.60	0.35	14	0.02	0.05	0.45
Hist. Th.	1,279	200	16.0	5.19	0.53	103	$\sim$ 0	0.27	$\sim$ 0
Hist. Uth.	1,717	224	15.3	4.95	0.51	140	$\sim$ 0	0.33	$\sim$ 0
Hist. Th., F. CO_2_	1,247	185	15.8	5.09	0.53	117	$\sim$ 0	0.29	$\sim$ 0
Hist. U-th. F. CO_2_	1,673	209	15.2	4.87	0.51	150	$\sim$ 0	0.34	$\sim$ 0

In order of the following columns: Number Density (N. Den.), Stand Biomass (C. Den.), mean tree height (Height), Leaf Area Index (LAI), Carbon Gini Coefficient (Gini). The last two columns are evaluations of the number density distribution across tree mass (see Fig. 1), with Pearson’s Chi-squared and Kolmogorov–Smirnov test. Aggregate, means, and goodness of fits, are estimated using the size-structure past the 16.69 kgC tree mass threshold corresponding to a survey minimum threshold of 7 cm dbh.

The historical simulations overestimated the unevenness of the distribution of biomass across the population within the stand in rank order of tree size (‘biomass inequality’). The biomass Gini coefficient is a measure of inequality: 0 implies perfect equality (e.g., the stand biomass is distributed across all size trees evenly, a 1:1 line in Fig. 1d) and 1 implies maximal inequality (e.g., a single tree with all the stand biomass). Gini inequality can be a useful metric when evaluating forest demography as there are clear differences between uneven-age and even-age stands^49^, and evaluating stand development^50^. In addition, Gini coefficients can also be indicative of vulnerability of forests to size-dependent disturbances such as windthrow^51^. Historical simulations had a significantly larger Gini coefficient than the observations, approximately 0.51 versus 0.35, respectively. The difference between the historical and fitted demography simulations implies that the growth and/or mortality rate depend differently on tree size than assumed in the model. This could be indicative of some demographic processes which are not represented in the simulations. For instance, targeted thinning of large or small trees could occur within a Sitka spruce plantation^44^. Smaller trees are more suppressed by competition^52^ or vulnerable to pests^53^, while larger trees are more vulnerable to windthrow^54^ and drought^55^.

The fitted-demography simulations represent a minimisation of the error arising from the modelled tree size-distribution. We initialised JULES-RED in 2015 with the observed tree-size distribution in 2018, thereby allowing for three years of dynamically modelled demography away from the original fit. The modelled forest remained close to key parameters of the measured forest: tree density (1348 ha^−1^ vs. 1248 ha^−1^), stand biomass (208 tC ha^−1^ vs. 219 tC ha^−1^), mean height (17.6 m vs 17.8 m) and leaf area index (LAI) (5.57 m^2^ m^−2^ vs. 5.59 m^2^ m^−2^). The remaining difference may be attributable to allometric relationships applied in both the derivation of the observed biomass and allometric assumptions in JULES-RED when aggregating to the community scale. Including modelled thinning increased the mean tree size and decreased the tree number and carbon density.

Compared to the observations, the thinned simulations had respectively 5.1–7.5% and 3.9–11% lower tree density and stand biomass (both Transient/T.-Fixed/F. CO_2_). Simulating removing a third of trees in 1998 decreased tree density by 25% in 2018 over unthinned historical simulations, indicating convergence of the unthinned and thinned tree densities. Thinning also reduced the stand biomass by 10–11% (T.-F. CO_2_). However, the remaining trees were marginally larger with thinning increasing the mean height by 3.9–4.1% and had more LAI by 4.2–4.9% (both T.-F. CO_2_). Thinning also increased the biomass Gini inequality of the forest by 3.8%. Long-term observations and empirical model comparisons of thinning vs unthinned stands agree that while both the stand density and biomass decreases, mean tree size and productivity for the remaining trees generally increase in the immediate decades after thinning^50,56,57^. LAI is expected to decrease directly after thinning and recover towards the LAI in unthinned stands^58,59^. Empirical model results have shown small increases of height of thinned stands over unthinned stands^60^. However, direct observations of Sitka spruce plantations have shown no significant relationship between thinning intensity and height growth^61^. It has been shown that in thinned plantations there is little difference between Gini coefficients (in terms of ‘growth inequality’) between unthinned and thinned stands^50^.

Compared to the observations, the unthinned simulations had 24–27% (F-T. CO_2_) greater tree density and 0.3–7.4% (F.-T. CO_2_) greater biomass. Including transient CO_2_ increased tree density and biomass by 2.6% and 7.0–8.0% (Unthinned/Uth. to Thinned/Th.), respectively, compared to the simulations with fixed 1973 CO_2_ concentrations. Transient CO_2_ also marginally increased mean tree height, LAI, and biomass Gini inequality, respectfully: 0.96–1.2% (Uth.-Th.), 1.6–2.1% (Uth.-Th.) and 0.94–0.95% (Th.-Uth.). Free-air CO_2_ enrichment (FACE) experiments are a useful measure of the impact of increased CO_2_ on forests^62^. Across multiple FACE experiment sites, there was an observed increase in forest biomass when enriched by CO_2_^63^. The Duke FACE experiment, an evergreen pine plantation (*Pinus taeda*) showed a clear increase in LAI at 200 ppm above the ambient^64^. However, the Oak Ridge FACE experiment in a deciduous broadleaved plantation *(Liquidambar styraciflua),* showed no statistically significant differences in LAI, height, and basal area distribution and canopy structure^65^ after 12 years of enrichment by an average of 152 ppm from the ambient CO_2_ concentrations (395 ppm).

### Evaluation of fluxes

**Figure 2:**
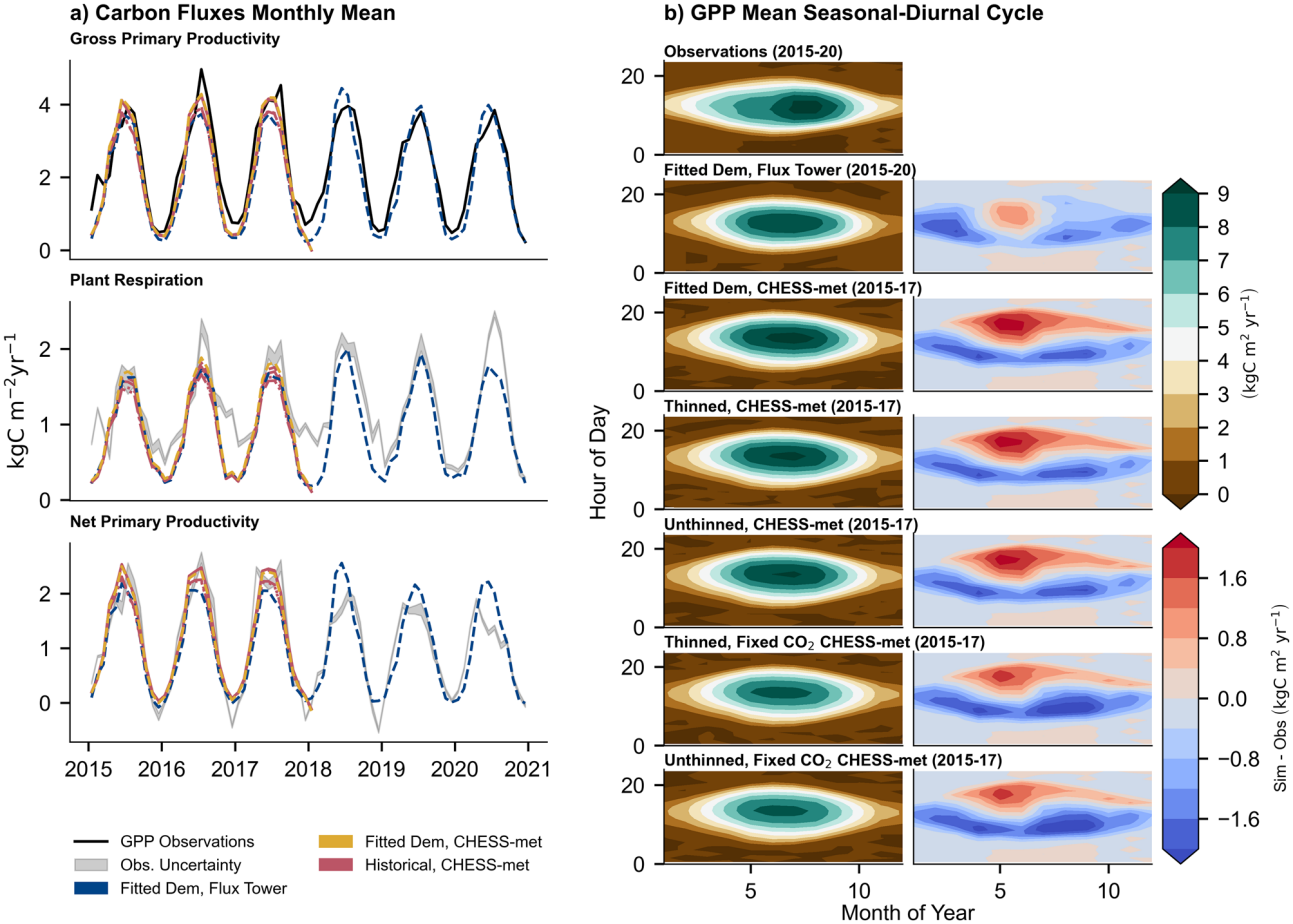
Observed and simulated carbon fluxes for the mature even-aged spruce stand in Harwood Forest. Panel (**a**) shows the monthly averages across the time-series (2015–20 for simulations using in-situ climate data and 2015–17 for Chess-Met 1 km gridded data) for GPP, tree respiration, and NPP. Black lines indicate the GPP observations while grey bands on tree respiration and NPP subplots indicate the uncertainty arising from partitioning of root respiration from the total soil respiration. Panel (**b**) shows both the seasonal (x-axis) and diurnal cycle (y-axis) of GPP, and difference between simulated GPP (red and blue scale) against observations. Similarly, to Fig. 1, solid and dashed dotted (Fixed 1973 CO_2_ concentration) olive lines are un-thinned historical simulations, while dashed and dotted (Fixed 1973 CO2 concentration) runs have been thinned.

The JULES-RED model simulations were able to reproduce the general seasonal cycle of monthly GPP (Fig. 2a). We explore the model vs. observations differences in seasonal and diurnal cycles (Fig. 2b). JULES-RED does a reasonable job of simulating the seasonal cycle, although simulations slightly overestimated summer GPP, while underestimating winter GPP. There were also differences between the daily peaks of GPP, with the peak occurring later in JULES-RED, especially during summer (positive Sim-Obs differences in Fig. 2b in the afternoon). The effect of including CO_2_ fertilisation is noticeable within the seasonal and diurnal cycles. Simulations with fixed 1973 CO_2_ concentration tended to have lower yearly and daily GPP peaks, significantly underestimating GPP in later summer. By 2015, thinning in 1998 had very little noticeable impact in seasonal-daily GPP. Simulations underestimated respiration between 2015 and 2017 when compared with inferred observations. Simulated monthly NPP performed relatively well for the first three years of observations. However, for the last three years (2018–20) the observed decline in GPP coupled with the greater observed tree respiration resulted in a modelled overestimation of NPP. It should be noted that the historical simulations were not able to make use of the full observed range for the fluxes (2015–17 compared with 2015–20).

The simulations running with fitted demography had negligible impact on the overall monthly errors of the carbon fluxes compared to historical simulations across the day or month. While using CHESS-met data to drive JULES-RED produced monthly GPP, tree respiration, and NPP values that agreed well with the monthly observations, the simulation with in-situ climate data had less monthly correlation with NPP and GPP but had a better overall fit to estimated respiration (Table 3). Using sub-daily in-situ meteorological drivers improved the comparison to the sub-daily observations for GPP. Including transient CO_2_ in the historical simulations decreased the error for the diurnal GPP and marginally for the tree respiration and NPP. Thinning had no significant reduction in error or improvement in correlation. In terms of the magnitude differences between the historical simulations, thinning only slightly increased the mean GPP and tree respiration by 0.4–0.5% (T.-F. CO_2_) and 3.0–3.2% (T.-F. CO_2_) respectively, while slightly decreasing the overall NPP by 1.5–1.7% (F.-T. CO_2_). CO_2_ fertilisation had a more significant increase on GPP, respiration, and NPP: 7.5–7.6% (Th.-Uth.), 5.8–6.0% (Uth.-Th.), and 8.8–9.0% respectively (Th.-Uth.). Finally, both processes together had a net effect on the GPP, tree respiration, and NPP of 8.0%, 9.1%, and 7.2%.

**Table 3:** The observed and simulated carbon fluxes for the stand at Harwood.

	GPP (kgC m^−2^ yr^−1^)					Tree Resp. (kgC m^−2^ yr^−1^)			NPP (kgC m^−2^ yr^−1^)		
	Mean	RMSE		R^2^		Mean	RMSE	Resp. R^2^	Mean	NPP RMSE	NPP R^2^
		S.D	M	S.D	M		M	M		M	M
Observations	2.28					1.21			1.07		
Fit. Dem., F.T	1.96	1.57	0.53	0.75	0.80	0.94	0.38	0.79	1.01	0.38	0.80
Fit. Dem., C	2.18	1.78	0.49	0.72	0.89	0.98	0.39	0.71	1.19	0.31	0.89
Hist. Th.	2.18	1.78	0.50	0.72	0.89	0.95	0.40	0.71	1.20	0.31	0.89
Hist. Uth.	2.17	1.78	0.50	0.71	0.89	0.92	0.42	0.71	1.22	0.31	0.89
Hist. Th., F. CO_2_	2.03	1.81	0.57	0.72	0.89	0.89	0.43	0.70	1.11	0.34	0.89
Hist. Uth. F. CO_2_	2.02	1.81	0.58	0.72	0.89	0.87	0.44	0.70	1.12	0.33	0.89

Mean for GPP, Tree respiration and NPP for overlap period with respect to the observations (2015–20), CHESS-met runs (Hist. & Fit. Dem., C.) only overlapped for 2015–17. Shows the goodness of fits for sub-daily 30 min intervals (S.D.) and monthly (M.) for the Root Mean Squared Error (RSME) and correlation (R^2^).

There are a lack of observational comparisons looking at the long-term effect of thinning (greater than 15 years) on carbon fluxes within Sitka spruce plantations. After 8 years, one study showed that thinning in a 100-year mixed forest stand (that included Sitka spruce) had little change on NEE because of the combined reduction in GPP and ecosystem respiration ^66^. A modelling study projected forest growth into 2100 across three European stands^30^. They found that under the control simulation GPP and autotrophic respiration decreased significantly by 2100, while the NPP sign varied across the sites from marginally negative to positive. Including CO_2_ fertilisation under future radiative forcing scenarios resulted in significant increases in carbon stock. The forest management implemented was different, as unlike this study, there was repeat thinning of 20–30% of basal area at regular intervals in the boreal site with complete harvesting and replanting at other sites. FACE experiments have shown a large response in GPP to elevated CO_2_ (> 150 ppm), a varied response of NPP and overall vegetation carbon across sites^63,67^. Nitrogen limitation provides one hypothesis for the varied responses of between GPP and NPP observed at FACE sites^68^. As a result, the latest LSMs now attempt to represent nutrient limitations and deposition^69,70^.

### Historical simulations

**Figure 3:**
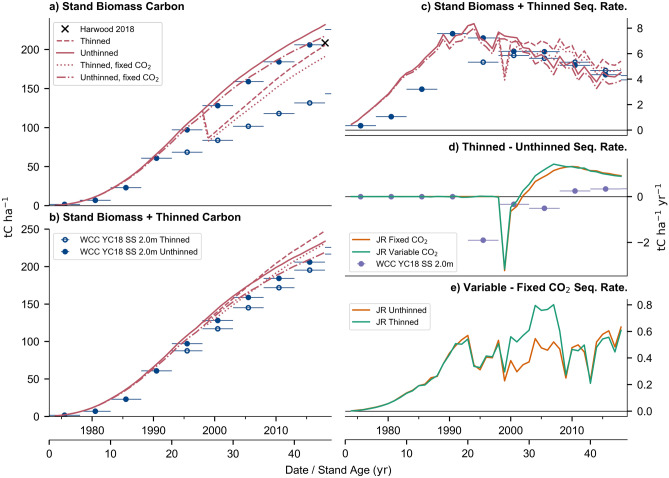
- Historical simulations of stand carbon stock. Panel (**a**) plots the cumulative stand biomass of both JULES-RED in red and Woodland Carbon Code (WCC) lookup table for YC18 Sitka spruce at 2.0 m in blue. The 2018 observed biomass is presented as a black cross. (**b**) Shows the cumulative carbon of both stand biomass and thinned material (i.e. for products) with panel (**c**) showing the rate of sequestration of both stand and thinning harvest rate. (**d**) Shows the difference between the thinned and unthinned simulations of panel (**c**) sequestration rate. (**e**) Shows the sequestration rate differences between simulations using varying and fixed (1973) CO_2_ concentrations.

Figure 3 shows the historical simulations across the 45-year period between 1973 and 2018 of vegetation carbon biomass and sequestration, with a direct comparison between controlled and simulated processes (e.g., unthinned vs thinned or fixed vs varying CO_2_ concentrations). As the stand in Harwood Forest is assessed as having a yield class of 18 m^3^ ha^−1^ yr^−1^ a comparison against the stand biomass and sequestration available from the WCC model results for both managed and unmanaged Sitka spruce YC18 was made. The closest projections to the 2018 observations of stand biomass were the simulations with unthinned fixed CO_2_, transient CO_2_ with thinning and the WCC unthinned projection.

The JULES-RED unthinned simulations agree well with the WCC results, with the unthinned fixed CO_2_ simulation being closest to the unthinned WCC growth curve (Fig. 3a,b). The WCC thinned results were much lower that the JULES-RED simulations, which is principally due to the different thinning strategy of repeated thinning after 15 years, compared to a third of trees at 25 years old. However, this difference illustrates the range of possible management intensities that could be employed. Figure 3.c shows the carbon sequestration rate of the stand biomass and thinning of both JULES-RED simulation and the WCC lookup table. JULES-RED simulates more sequestration in young stands than the WCC curve, but the peak year, magnitude of the peak, and subsequent decline of the sequestration rate are similar in both models.

Figure 3d shows the difference between the thinned and unthinned simulations and results from the WCC model. Imposing a tree thinning of a third in 1998 resulted in greater modelled growth rates for the remaining trees, with more vegetation carbon sequestered post-thinning when compared against the un-thinned simulations (Fig. 3b–d). In comparison, thinning only sequesters more carbon in the WCC curve after a stand age of 35. Assuming that the carbon in the thinnings was put into a non-decaying harvest carbon pool resulted in greater total net carbon sequestration over the stand’s life cycle (Fig. 3b). However, this assumption is simplistic as products from using thinned wood have a variety of possible turnover rates^71^.

There was steady divergence in the magnitude of stand biomass between the fixed and varying CO_2_ concentration simulations. Early age effects on juvenile trees resulted in less time to reach the peak sequestration rate (Fig. 3e), before steadily becoming more linear and variable as the stand aged. In comparison to the FACE experiments, it has been observed that CO_2_ fertilisation may have a transient impact on juvenile trees in younger stands^63^. For two-year old Sitka spruce, it has been observed that an increase of 250 ppm from the ambient caused an increase of growth of about 9.8% when not water limited^72^. However, these are not direct comparisons as all transient CO_2_ simulations initially started at 1973 concentrations and diverge, not an abrupt increase. By stand maturity, there is a hypothesis that any increases in carbon biomass seen caused by CO_2_ fertilisation is entirely transient^62,65^, with ecosystem respiration offsetting any gains^73^, this differs from the eventual biomass achieved in the JULES-RED model.

## Discussion

Including thinning and CO_2_ fertilisation effects influenced the demography, fluxes, and biomass growth within the stand. Including thinning was significant in reducing the overall error comparing model results with the 2018 observations of size-structure. Thinning reduced the tree and biomass densities, increased mean tree size and LAI of the stand (Fig. 1 and Table 2). This result is generally consistent with observed comparisons and empirical simulations of thinned and unthinned Sitka spruce stands^50,56–60^. While including CO_2_ fertilisation had little impact in improving the fits to the 2018 observations, the process reduced the modelled and observed difference of overall mean stand characteristics and the effect had a greater impact on eventual tree and carbon density. As the FACE experiments have shown^65^, disentangling the impact of raised CO_2_ concentrations on demography from age is difficult. However, in FACE experiments there were observations of increased carbon biomass and LAI^63^. The net effect of both processes resulted in increases of tree density, biomass, and tree size. This outcome is possibly very dependent on the form of forestry management assumed, as more intense modelled management regimes have shown a large reduction in stand biomass from repeat thinnings^30,45^. The fitted simulations demonstrated that the model allometric relationships provided a reasonable comparison for the total and mean properties when compared with the observations, such as total biomass density, LAI, and mean height. All the historical simulations overestimated the number of small and large trees and therefore the biomass inequality within the stand by 2018. This overestimation could be because some size-dependent mortality and/or competitive processes are ignored^44,52–55^. There is clear future potential for LSM to fully utilise representation of demography to improve these size-dependent processes.

All JULES-RED simulations were able to replicate the in-situ mean monthly carbon fluxes of GPP, NPP while underestimating tree respiration (Fig. 2 and Table 3). JULES-RED was unable to fully capture the increase in respiration and decline in NPP after 2018. When compared to the simulation of diurnal GPP results, model error and correlation against observations, respectively increased and decreased. Simulations driven by meteorological observations from the flux tower resulted in the lowest error diurnally. In comparison, using the fitted demography produced very little reduction in error when compared against the effect on the historic simulations. Other potential improvements can come from using improved PFT traits for photosynthesis and water demand. For instance, the choice of using the van Genuchten curve may underestimate the impact of water limitation on plant productivity^74^. The impact of earlier thinning had a negligible effect on modelled carbon fluxes. There are not very many direct observations of the long-term effect thinning has on GPP with similar management in Sitka spruce stands^75,76^. The inclusion of transient CO_2_ increased GPP and NPP and decreased the model-observation GPP error. While the increase in GPP is expected, FACE experiments suggest a more varied response for NPP^63,67^, possibly due to carbon to nitrogen limitation^68^ and increased ecosystem respiration^73^. LSM do have the scope to include the impact of nitrogen on carbon fluxes^70^ and the changes in carbon allocation and respiration response to raised CO_2_^77^.

The unthinned fixed CO_2_ historical simulation by JULES-RED (Fig. 3) broadly agrees with the growth curve of the WCC model for a YC18 unthinned Sitka spruce stand. This is a useful comparison to make as it demonstrates that the model can replicate the current empirical understanding of forestry stand growth within the UK, when CO_2_ fertilisation is ignored. The comparison between thinned simulations and WCC shows the range of possible management intensities that could be employed. Including both processes, thinning and CO_2_ fertilisation in the simulations produces stand biomass estimates that were close to the 2018 observations. An assumption that woody products from thinning do not decay, results in overall more net carbon being sequestered compared to the unthinned scenario. This is unrealistic, as carbon sequestered from woody products can potentially have a range of lifetimes^71^. In comparison to the unthinned runs, thinning had less sequestration directly after followed by more sequestration a few years after. The WCC results demonstrate that having more management can result in a delay to having more carbon sequestered over a unthinned stand. Compared to the fixed 1973 concentration, transient CO_2_ resulted in an age dependent acceleration of carbon sequestered, followed by more variable linear increase in carbon sequestered. Other studies have shown that the impact of raised CO_2_ concentrations on juvenile Sitka spruce or stands can cause significant increases carbon accumulation^63,72^.

Many governments are planning on using afforestation to meet their NDCs^2^. Accurate estimation of present and future carbon stocks, emissions, and sequestration is required for implementation of robust land-use policy. The recent introduction of forest demography within global LSMs is making these models much more fit for this purpose. In this study, we have demonstrated this by using the JULES-RED model to simulate the growth of a British upland Sitka spruce plantation. Currently, models used to estimate carbon credits and afforestation contributions to NDCs do not account for CO_2_ fertilisation and are likely to underestimate CO_2_ sequestration. We have also shown that JULES-RED can incorporate management impacts such as thinning, which is an essential addition. Including both a single thinning operation and the increasing CO_2_ concentration in the model increased the overall carbon sequestered between 1973 and 2018 by around 28.6 tC ha^−1^ or 13% in JULES-RED over the control simulation. The true impact of both effects is uncertain and other neglected processes may cause current sequestration projections to be overestimates, such as the impact of future increases in drought.

The inclusion of demography into LSMs allows a better link between site-level and global-scale modelling^25^. As demonstrated in this study, this enables bottom-up constraints from the site level. Including top-down constraints already applied to gridded LSM^78^, can help to give users even more confidence in model projections. As we transition toward Net Zero, we increasingly need to know how efficient and resilient specific forestry mitigation actions will be. Demographic LSMs have the potential to provide a consistent tool that can be used to inform local land-use decisions—“which trees to plant where?”—and to inform climate negotiations—“how will NDCs affect atmospheric CO_2_ and global mean temperature?” To make full use of the potential of LSM modelling to provide answers for policy relevant questions, further model developments need to be made. It is imperative that more measurements of vegetation dynamics and demographics are available for comparison to demographic models: tree density, mortality, fecundity, and the size-structure. Surveys do not necessarily have to encompass a large area to evaluate the demography of the model. For example, the observations used here are from 10 plots totalling approximately 0.2 ha.

### Supplementary Information

Supplementary Information.

## Data Availability

The version of the JULES-RED model used in this paper is available from the Met Office code repository (code.metoffice.gov.uk), applying for access is done via an online form: http://jules-lsm.github.io/access_req/JULES_access.html (accessed 03/02/2023). JULES-RED is a test branch labelled: r24142_test_vn7.0_add_red_sci_vn1.1, along with the model suite for running at Harwood is provided as a rose suite: u-cn548 on the repository. The CHESS-met^31^ dataset can be found through the link: https://doi.org/10.5285/2ab15bf0-ad08-415c-ba64-831168be7293, while the HWSD soil van Genuchten parameters ancillaries for the UK are detailed in Pinnington et al.^47^. Observations and JULES-RED outputs are stored in a data repository^79^: https://doi.org/10.5281/zenodo.7603502.
