# Supplementary material for: Modelling the impact of forest management and CO2-fertilisation on growth and demography in a Sitka spruce plantation
Source: Sci Rep. 2023 Aug 18;13:13487. doi: 10.1038/s41598-023-39810-2 (PMC10439122; doi:10.1038/s41598-023-39810-2)
Supplement: Supplementary file 1 — Supplementary Information. [file 41598_2023_39810_MOESM1_ESM.pdf]

Modelling the impact of forest management and CO_2-fertilisation on growth and demography in a Sitka spruce plantation

Arthur P. K. Argles[1,2], Eddy Robertson[1], Anna B. Harper[2], James I. L. Morison[3], Georgios Xenakis[4], Astley Hastings[5], Jon Mccalmont[5,6], Jon R. Moore[2], Ian J. Bateman[7], Kate Gannon[7], Richard A. Betts[1,8], Stephen Bathgate[4], Justin Thomas[5], Matthew Heard[9], and Peter M. Cox[2]

[1]Met Office Hadley Centre, FitzRoy Road, Exeter, Devon, EX1 3PB, UK

[2]Department of Mathematics and Statistics, Faculty of Environment, Science and Economy, University of Exeter, Exeter, EX4 4QE, UK

[3]Forest Research, Alice Holt Lodge, Surrey, GU10 4LH, Farnham, UK

[4]Forest Research, NRS, Roslin, Midlothian EH25 9SY, UK

[5]School of Biological Sciences, University of Aberdeen, King's College, Aberdeen AB24 3FX, UK

[6]Department of Biosciences, Faculty of Health and Life Sciences, University of Exeter, Streatham Campus, Rennes Drive, Exeter EX4 4RJ, UK

[7]Land, Environment, Economics and Policy Institute (LEEP), Department of Economics, University of Exeter Business School, Exeter, UK

[8]University of Exeter Global Systems Institute, Exeter, EX4 4QE, UK

[9]The National Trust, Heelis, Kemble Drive, Swindon, SN2 2NA UK

Supplementary Information: JULES-RED Model Description

The Joint UK Land Environment Simulator (JULES) Land Surface Model (LSM) is used to simulate the bio-geophysics of the land surface[1,2] across a landscape represented by tiles. JULES calculates the photosynthetic pathways for C_3 and C_4 using the Collatz et al., 1991[3] and , 1992[4] models, respectively. In the JULES model, the soil hydrology is simulated using a discretisation of the Richards equation and the van Genuchten[5] water retention and hydraulic conductivity curves. As outlined in Clark, D. et al., 2011[2], tile carbon pools and litter fluxes were estimated for wood, leaf, and root, using Plant Functional Type (PFT) tile allometric and litter production rate equations. These relationships have been improved with evaluations against remotely sense and site comparisons, with more specific PFTs, such as Needleleaf Evergreen Tree (NET), being introduced[6,7]. JULES calculates the carbon and water fluxes at a half hourly timestep, with the vegetation dynamics (tree carbon, height and LAI), being updated daily.

The Robust Ecosystem Demography (RED) DGVM has been coupled into the JULES LSM[8]. JULES-RED partitions the number density, n (kgC^{-1} m^{-2}), of each Plant Functional Type (PFTs) into mass, m (kgC), size classes and updates the size-structure by using equation (S1), a Fokker-Planck continuity equation of plant growth, g (kgC yr^{-1}), and mortality, γ (yr^{-1}):

$$\frac{\partial n}{\partial t} - \frac{\partial}{\partial m}[n\,g] = -\gamma n. \tag{S1}$$

Grid-box vegetation coverage, or tile fraction, is normally estimated by taking the integral of the product of number density and crown area, a (m^2). However, this integral can potentially exceed the grid-box area when the number density is sufficiently high, such as in a Sitka spruce plantation[9]. Therefore, we have implemented a non-restricted "crown-area" fraction, ν_{CA}, (equation (S2)) which is not truncated to 1:

$$\nu_{\mathrm{CA}} = \int_{m_0}^{\infty} n\,a\,dm\,, \qquad a = a_0\left(\frac{m}{m_0}\right)^{0.5}. \tag{S2}$$

The "top-down" or grid-box vegetation fraction for PFT i is given in equation (S3):

$$\nu_i = \begin{cases} \nu_{\mathrm{CA},i}, & \sum_j \nu_{\mathrm{CA}\,j} \le 1 \\ \nu_{\mathrm{CA},i} - \nu_{>1,i}, & \sum_j \nu_{\mathrm{CA}\,j} > 1 \end{cases}, \tag{S3}$$

where $\nu_{>1}$ is the canopy reduction from ν_{CA} to be seen from above the canopy. The necessary reduction of $\nu_{>1,i}$ for each PFT, is estimated from the cumulative PFT sum in order of ascending height in the mass classes and PFT dimensions, until the total difference ($\sum \nu_{\mathrm{CA},i} - \nu_{>1,i}$) across PFTs drops below 1. RED takes JULES inputs of carbon assimilate density (P), which in JULES-RED is the difference of estimated NPP (Π_{NPP}) and the local litterfall (Λ_{LLF}) multiplied by the grid-box coverage (for PFT grid-box density), as demonstrated by equation (S4):

$$P = \nu(\Pi_{\mathrm{NPP}} - \Lambda_{\mathrm{LLF}}). \quad \text{(S4)}$$

The addition of canopy closure results in a simplistic method of limiting the overall growth rate as coverage is truncated to 1. Equation (S5) shows the recruitment dynamics. Recruitment assumes a fraction (α) of the PFT assimilate is devoted to seedling reproduction:

$$\frac{\alpha P}{m_0}\left(1 - \sum c_{i,j}\nu_{\mathrm{CA},j}\right) = n_0 g_0, \quad \text{(S5)}$$

where the number of successful recruits is limited by the amount of space not occupied by dominant PFTs. The structural assimilate (G) is then partitioned among the mass classes through Metabolic Scaling Theory, equation (S6) [10,11]:

$$G = (1-\alpha)P = \int_{m_0}^{\infty} n\, g\, dm\,, \qquad g = g_0\left(\frac{m}{m_0}\right)^{0.75}, \quad \text{(S6)}$$

where at each time-step we invert equation (S6) for the boundary growth rate g_0. For mortality, JULES-RED assumes a fixed size invariant background mortality rate. JULES-RED is ultimately designed to be as parsimonious as possible while still including a realistic representation of size, so the model is not as complicated as other DGVMs[12,13]. As shown by equation (S7), JULES-RED uses allometric relationships to determine the PFT height (h) and balanced LAI (l) used throughout the wider model (e.g. determining photosynthesis, phenology, or surface roughness):

$$h = h_0\left(\frac{m}{m_0}\right)^{0.25}, \quad l_{\mathrm{bal}} = l_{\mathrm{bal,0}}\left(\frac{m}{m_0}\right)^{0.25}. \quad \text{(S7)}$$

To simulate JULES-RED, we require radiative and meteorological forcings: air pressure, air temperature, specific humidity, precipitation, wind speed, downward short and long wave radiation. To run with daily data JULES-RED also requires the daily temperature range. We also require soil property variables to run the van Genuchten model.

For simulating small initial trees an additional lower mass class of $m_0 = 0.1\ \mathrm{kgC}$ (normally $1.0\ \mathrm{kgC}$) was added onto the lowest NET mass class, and we simulate 30 geometrically spaced mass classes up to 50,000 kgC (standard configuration maximum tree mass class in RED). Additionally, the allometric parameters for $a_0 = 0.23\mathrm{m}^2$, $h_0 = 2.89\mathrm{m}$, $l_{\mathrm{bal,0}} = 0.8\ \mathrm{m}^2\mathrm{m}^{-2}$. Allometry follows a power-law relationship with mass (equation (S6)), with the power for height and balanced LAI (l_{bal}) being 0.25. Generally, these power-law allometries, especially for height, are less accurate for smaller than for larger trees[11]. For NET, we assume a small, reseed fraction of $\alpha = 0.005$, this is to mimic the low recruitment rate of trees in these dense planted stands of young Sitka spruce trees[14]. We assume a baseline mortality rate of $\gamma = 0.01\ \mathrm{yr}^{-1}$, this value is lower than the mean mortality rate for Sitka spruce presented in the Forestry Commission yield management booklet for an un-thinned stand of ages between 35-49[15].

References

[1] Best, M. J. *et al.* The joint uk land environment simulator (jules), model description – part 1: energy and water fluxes. *Geosci. Model Dev.* **4**, 677–699 (2011)

[2] Clark, D. B. *et al.* The joint uk land environment simulator (jules), model description – part 2: carbon fluxes and vegetation dynamics. *Geosci. Model Dev.* **4**, 701–722 (2011)

[3] James Collatz, G., Ball, J. T., Grivet, C. & Berry, J. A. Physiological and environmental regulation of stomatal conductance, photosynthesis and transpiration: a model that includes a laminar boundary layer. *Agric. For. Meteorol.* **54**, 107–136 (1991)

[4] Collatz, G., Ribas-Carbo, M. & Berry, J. A. Coupled photosynthesis-stomata1 conductance model for leaves of c4 plants. *Funct. Plant Biol.* **19**, 519–538 (1992)

[5] van Genuchten, M. T. A closed-form equation for predicting the hydraulic conductivity of unsaturated soils. *Soil Sci. Soc. Am. J.* **44**, 892–898 (1980)

[6] Harper, A. B. *et al.* Improved representation of plant functional types and physiology in the joint uk land environment simulator (jules v4.2) using plant trait information. *Geosci. Model Dev.* **9**, 2415–2440 (2016)

[7] Harper, A. B. *et al.* Vegetation distribution and terrestrial carbon cycle in a carbon cycle configuration of jules4.6 with new plant functional types. *Geosci. Model Dev.* **11**, 2857–2873 (2018)

[8] Argles, A. P. K. *et al.* Robust ecosystem demography (red version 1.0): a parsimonious approach to modelling vegetation dynamics in earth system models. *Geosci. Model Dev.* **13**, 4067–4089 (2020)

[9] Hale, S. E. Light regime beneath sitka spruce plantations in northern britain: preliminary results. *For. Ecol. Manage.* **151**, 61–66 (2001)

[10] Brown, J. H., Gillooly, J. F., Allen, A. P., Savage, V. M. & West, G. B. Toward a metabolic theory of ecology. in *Ecology* vol. 85 1771–1789 (Ecological Society of America, 2004).

[11] Niklas, K. J. & Spatz, H.-C. Growth and hydraulic (not mechanical) constraints govern the scaling of tree height and mass. *Proc. Natl. Acad. Sci.* **101**, 15661–15663 (2004)

[12] Fisher, R. A. *et al.* Vegetation demographics in earth system models: a review of progress and priorities. *Glob. Chang. Biol.* vol. 24 35–54 at https://doi.org/10.1111/gcb.13910 (2018)

[13] Argles, A. P. K., Moore, J. R. & Cox, P. M. Dynamic global vegetation models: searching for the balance between demographic process representation and computational tractability. *PLOS Clim.* **1**, e0000068 (2022)

[14] Mair, A. R. Dissemination of tree seed sitka spruce, western hemlock and douglas fir. *Scottish For.* **27**, 308–314 (1973)

[15] Matthews, R. W., Jenkins, T. A. R., Mackie, E. D. & Dick, E. . *Forest Yield A Handbook On Forest Growth And Yield Tables For British Forestry*. (2016).
